# Adverse childhood experiences and adult dental care utilization in the United States: Variation by race and ethnicity

**DOI:** 10.1371/journal.pone.0332880

**Published:** 2025-09-30

**Authors:** Alexander Testa, Karyn Fu, Dylan B. Jackson, Rahma Mungia, Thomas W. Oates, Jason M. Nagata, Kyle T. Ganson

**Affiliations:** 1 Department of Management, Policy and Community Health, School of Public Health, University of Texas Health Science Center at Houston, Houston, Texas, United States of America; 2 Rice University, Houston, Texas, United States of America; 3 Department of Population, Family, and Reproductive Health, Johns Hopkins Bloomberg School of Public Health, Baltimore, Maryland, United States of America; 4 Department of Periodontics, School of Dentistry, University of Texas Health Science Center at San Antonio, San Antonio, Texas, United States of America; 5 Department of Advanced Oral Sciences and Therapuetics, School of Dentistry, University of Maryland, Baltimore, Maryland, United States of America; 6 Department of Pediatrics, University of California, San Francisco, California, United States of America; 7 Factor-Inwentash Faculty of Social Work, University of Toronto, Toronto, Ontario, Canada; Medical College of Wisconsin, UNITED STATES OF AMERICA

## Abstract

Adverse childhood experiences (ACEs) are associated with negative health outcomes, including poorer oral health. While research has shown that ACEs are associated with lower dental care utilization, most studies focus on childhood and adolescence, with limited attention to their long-term impact in adulthood or differences across racial and ethnic groups.. This study examines the relationship between ACE exposure and past-year dental care use in adulthood and assesses racial/ethnic differences in this association. Using data from the 2020 Behavioral Risk Factor Surveillance System (BRFSS), we analyzed a sample of 88,728 adults from 21 states and the District of Columbia. The primary outcome was any past-year dental care use. ACEs were summed into a composite score. Multivariable logistic regression models assessed the relationship between ACEs and past-year dental care use, with multiplicative interaction terms used to examine racial/ethnic differences in the observed association. Overall, 63.2% of adults reported any past-year dental care use. Higher ACE exposure was associated with lower odds of dental care use (adjusted odds ratio [aOR] = 0.95, 95% CI: 0.93–0.97, *p* < .001). The negative association between ACEs and dental care use was strongest for non-Hispanic White respondents, whereas the relationship between ACEs and dental care was attenuated for non-Hispanic Black and Hispanic respondents. These findings expand knowledge on the association of ACEs with dental care use in adulthood and how this relationship may vary across the population.

## Introduction

Adverse childhood experiences (ACEs)—i.e., potentially traumatic events occurring before age 18, including abuse (i.e., physical, sexual, emotional abuse), neglect (i.e., physical, emotional neglect), or household dysfunction (i.e., domestic violence, parental incarceration, parental separation/divorce) [1]—are a serious public health issue due to their potential to lead to poor health outcomes and high societal and economic costs over the life course [2–5]. Emerging research demonstrates that ACEs are linked to poorer oral health and lower uptake of dental care [6–8]. However, much of this research has focused on childhood and adolescence [6,7], a developmental period where caregivers or parents heavily influence dental care patterns, and rates of dental care utilization are nearly 20 percentage points higher (80.9% for children ages 1–17) [9] than adulthood (62.7% for adults ages 18–64) [10]. Even so, far less is known about the long-term implications of ACEs on dental care use during adulthood [8].

Theoretically, ACEs may reduce adult dental care use for several reasons. First, ACEs are associated with lower dental care use in childhood and adolescence [6,7], potentially establishing patterns that set the stage for lower dental care use that persist into adulthood [11]. Second, ACEs may erode trust in medical professionals and social institutions, leading individuals to avoid engagement with dental practitioners [12,13]. Relatedly, ACEs often involve traumatic experiences that can heighten fear and anxiety in clinical settings, including dental offices. Because receiving dental care requires close physical proximity to providers, it may trigger memories of past abuse or neglect, leading some individuals to avoid care out of fear of retraumatization [11]. Finally, the cumulative disadvantage framework posits that negative experiences in early life can set off a chain of additional adversities that build up over time [14,15], ultimately resulting in poorer outcomes in adulthood, such as lower socioeconomic status (SES), [16,17] which is a well-established barrier to dental care access and utilization [18,19].

Regarding the relationship between ACEs and adult dental care, multiple studies using data from the 2010 Behavioral Risk Factor Surveillance System (BRFSS) have documented an association between higher ACE exposure and a lower likelihood of dental care use [20,21]. Relatedly, Crouch et al. found that individuals with four or more ACEs were approximately 2.8 times more likely to report not having a dental care visit in the past year using the 2016 South Carolina BRFSS [22]. Testa et al. analyzed data from the 2017–2021 Pregnancy Risk Assessment Monitoring System (PRAMS) in North Dakota and South Dakota, reporting that women with higher ACE exposure were about 25% less likely to have had a dental cleaning during pregnancy [23].

However, the extant research on ACEs and dental care remains limited in two key ways, which the current study seeks to expand. First, the limited research on ACEs and dental care in adulthood uses data over 15 years old [20,21] or are from a single or small set of states, [22,23] thereby limiting an understanding of the generalizability between ACEs and dental care in a broader, contemporary sample. Second, because of the use of narrow samples, prior research has been underpowered to assess heterogeneity in the relationship between ACEs and dental care across key features associated with less dental care utilization, such as one’s self-identified racial and ethnic backgrounds [10].

To be sure, there are reasons to expect the association between ACEs and dental care use in adulthood to vary across race/ethnicity. Within the cumulative disadvantage framework, ACEs are theorized to be potentially consequential for marginalized groups by depleting the already scarce social resources available, thus furthering the likelihood of negative events occurring over time [1,14,24]. Therefore, ACEs may exacerbate pre-existing disparities in dental care, potentially amplifying the already large racial inequities in dental care use for Black and Hispanic populations [10,15]. In contrast, the disadvantage saturation hypothesis posits that populations already at elevated risk for poor dental care—such as racial and ethnic minorities—may experience fewer additional consequences from high ACE exposure [15,24,25]. This is because these populations are already subject to substantial structural and social adversities that create barriers to dental care [26–28], which may dilute the influence of childhood adversities over the life course.

The current study uses recent data from 21 states and the District of Columbia that included data on both ACEs and past-year dental care use from the 2020 BRFSS to examine two key questions: (a) what is the relationship between ACEs exposure and past-year dental care use in adulthood? and (b) how does the relationship between ACE exposure and past-year dental care use in adulthood vary by self-reported race and ethnicity?

## Methods

### Data

Data included in this study were from the 2020 Behavioral Risk Factor Surveillance System (BRFSS), an annual national cross-sectional telephone survey conducted by the Centers for Disease Control [29]. The BRFSS survey employs a random digit dialing method for both cellular and landline phones to administer a questionnaire on health-related risk behaviors, chronic health conditions, preventive healthcare practices, and participant sociodemographic characteristics. Respondents are noninstitutionalized, civilian adults 18 years or older, living in a private residence or college housing in all 50 states, the District of Columbia, and three United States territories [29]. While the BRFSS survey consists of a standardized core questionnaire administered to all states, optional “opt-in” modules and state-added questions are administered in a subset of participating jurisdictions [29].

In 2020, the BRFSS had a 47.9% response rate, completing 401,958 interviews [30].The analytic sample for the current study consists of 88,728 individuals from 21 states and the District of Columbia (the capital city and federal district of the United States) who participated in the “Adverse Childhood Experiences” optional state module (refer to Appendix A for details of the sample selection procedures; Appendix B for the number of individuals by jurisdiction). The BRFSS data are publicly available and were fully anonymized at the time of the analysis. Accordingly, the BRFSS data did not qualify as human subjects research and was exempt from institutional review board review. Missing data were handled via listwise deletion.

### Dependent variable

*Past-year dental care use* is based on a question asking, “Including all types of dentists, such as orthodontists, oral surgeons, and all other dental specialists, as well as dental hygienists, how long has it been since you last visited a dentist or a dental clinic for any reason?” BRFSS respondents were then asked to pick one of the following responses: Never, less than 12 months ago, 1 year but less than 2 years ago, 2 years but less than 5 years ago, 5 or more years ago, don’t know/not sure, refused [31]. Using these responses, respondents were coded as a value of 1 if they reported a dental care visit within the past year and 0 if it had been over one year since the last dental care visit. Individuals who responded “don’t know/not sure” or refused were coded as missing values. This coding scheme was selected as it ensures adequate cell sizes for the moderation analysis of the number of ACEs by racial/ethnic subgroups.

### Independent variable

*Adverse childhood experiences* are based on a series of questions from the state optional module “Adverse Childhood Experiences,” where respondents answered yes or no to retrospective questions on ACEs occurring before age 18 years. The ACE measures included were as follows: (a) household mental illness, (b) household alcoholism, (c) household illegal drug use, (d) household incarceration, (e) parents divorced or separated, (f) household domestic violence, (g) physical abuse, (h) verbal abuse, and (i) sexual abuse – measured as any sexual abuse before age 5. Refer to Appendix C for details [31]. ACEs were summed into a single scale ranging from 0 to 9. Responses were top-coded at seven or more for analysis to preserve adequate cell size across all ACE levels [32–34].

### Moderating variable

*Race and Ethnicity* are based on respondents’ self-reports of their identified racial/ethnic groups, based on two questions: (1) which one or more of the following would you say is your race?; (2) Are you Hispanic, Latino/a, or of Spanish origin? Based on responses, the BRFSS data created a pre-computed race/ethnicity grouping variable with the following response options: non-Hispanic White, non-Hispanic Black, Hispanic, non-Hispanic Multiracial, or non-Hispanic Other Race (*_racegr4*). For this analysis, we retained respondents who identified as Hispanic only, non-Hispanic Black, and non-Hispanic White. Respondents not belonging to these racial/ethnic groups were excluded from the analytic sample due to small cell sizes that did not permit an assessment of heterogeneity by levels of ACE exposure.

### Control variables

Control variables including sociodemographic features that may be related to dental care use, including respondent *age* (18–24, 25–34, 35–44, 45–54, 55–65, or 65+), *sex* (male, female), *marital status* (married, divorced/separated, widowed, never married, or member of an unmarried couple), *educational attainment* (less than high school, high school graduate, some college, or college graduate), *child in home* (yes or no), *military veteran* (yes or no), *household income* ($ < 10,000, $10,000 - $14,999, $15,000 - $19,999, $20,000 - $24,999, $25,000 - $34,999, $35,000 - $49,999, $50,000 - $74,999 or ≥ $75,000), and *health insurance* (yes or no).

### Analytic approach

Weighted percentages for the full analytic sample and each racial/ethnic group were calculated. Multivariable logistic regression was used to assess the relationship between the number of ACEs and past-year dental care use. Three separate models were estimated: (1) bivariate relationship, (2) controlling for race/ethnicity, and (3) all controls. Next, we examined how the relationship between the number of ACEs and past-year dental care use differs between racial/ethnic groups by including a multiplicative interaction term between race/ethnicity and the number of ACEs in the bivariate model and model with controls. Analyses incorporated BRFSS survey weights using the *svy* command in STATA v.18.5 (StataCorp). Statistical significance was determined at the *p* < .05 threshold (two-tailed).

## Results

Of the 88,728 adults in the sample, 63.2% reported having had a dental visit in the past year. Non-Hispanic White respondents had the highest percentage of past-year dental care use (66.3%), followed by non-Hispanic Black respondents (61.5%), then Hispanic respondents (53.7%). Results of a Chi-squared test illustrate that the bivariate relationship between past-year dental care use and race and ethnicity is statistically significant (p < .001). In the analytic sample, the mean number of ACEs is 1.78 ACEs. The results of an ANOVA test revealed no statistically significant differences in the mean number of ACEs between respondent race/ethnicity (see Table 1).

**Table 1:** Weighted Summary Statistics of the Analytic Sample from the Behavioral Risk Factor Surveillance System, 2020 (N = 88,728).

	Full Sample (N = 88,728)	Hispanic (N = 7,333)	Non-Hispanic Black (N = 8,328)	Non-Hispanic White (N = 73,067)	*p*-value^a^
**Variable**					
*Dental Visit in Past Year*					<0.001
No	36.8%	46.3%	38.5%	33.7%	
Yes	63.2%	53.7%	61.5%	66.3%	
*Race/Ethnicity*					
Hispanic	8.2%				
Non-Hispanic Black	9.3%				
Non-Hispanic White	81.7%				
Number of ACEs (x [SD])	1.78 [1.93]	1.75 [1.99]	1.79 [1.91]	1.79 [2.01]	0.789
* Age*					<0.001
18-24	10.1%	16.5%	9.4%	8.4%	
25-34	15.7%	19.9%	18.2%	14.0%	
35-44	16.9%	20.8%	19.2%	15.2%	
45-54	16.5%	17.4%	19.1%	15.7%	
55-64	18.1%	14.4%	16.8%	19.5%	
65+	22.7%	11.0%	17.4%	27.3%	
*Sex*					<0.001
Female	51.2%	50.2%	54.4%	50.8%	
Male	48.8%	49.8%	45.6%	49.2%	
*Marital Status*					<0.001
Married	52.6%	46.5%	34.6%	58.2%	
Divorced or Separated	14.4%	15.1%	17.1%	13.6%	
Widowed	7.0%	3.1%	6.8%	8.1%	
Never Married	21.3%	27.7%	37.7%	15.9%	
Member of an Unmarried Couple	4.7%	7.6%	3.8%	4.1%	
*Educational Attainment*					<0.001
Less than High School	11.9%	28.6%	10.3%	7.5%	
High School Graduate	28.0%	28.3%	30.4%	27.3%	
Some College	33.1%	27.8%	34.9%	34.3%	
College Graduate	27.0%	15.3%	24.3%	30.9%	
*Child in Home*					<0.001
No	63.9%	48.9%	61.1%	68.8%	
Yes	36.1%	51.1%	38.9%	31.2%	
*Veteran Status*					0.417
No	88.4%	93.5%	87.7%	87.1%	
Yes	11.6%	6.5%	12.3%	12.9%	
*Household Income*					<0.001
Less than $10,000	4.6%	8.4%	8.8%	2.6%	
$10,000 - $14,999	4.5%	7.6%	5.4%	3.4%	
$15,000 - $19,999	7.5%	13.3%	11.0%	5.1%	
$20,000 - $24,999	8.9%	12.1%	11.0%	7.5%	
$25,000 - $34,999	9.8%	10.8%	12.1%	9.0%	
$35,000 - $49,999	13.7%	14.1%	14.8%	13.3%	
$50,000 - $74,999	16.2%	13.7%	13.5%	17.4%	
$75,000 or more	35.0%	20.1%	23.4%	41.7%	
*Health Insurance*					<0.001
No	14.9%	34.1%	15.6%	9.3%	
Yes	85.1%	65.9%	84.4%	90.7%	

^a^p-value represents the results from Chi-Squared tests. The table presents weighted percentages.

*Abbreviations:* ACEs = Adverse Childhood Experiences.

Table 2 reports the results of multivariable logistic regression analyses. In a bivariate model, every additional ACE experienced by an individual is associated with an 8% lower odds of past-year dental care use (adjusted odds ratio [aOR] =.92, 95% Confidence Interval [CI] =.90 −.93, p < .001). The results in Model 2 demonstrate that this association remains unchanged after controlling for respondent race/ethnicity (aOR =.92, CI = .90 –.93, p < .001). Moreover, in Model 2, the results show that the odds of past-year dental care use are lower amongst non-Hispanic Black respondents (aOR =.81, CI = .74 –.90, p < .001) and Hispanic respondents (aOR =.59, CI = .52 –.66, p < .001), compared to non-Hispanic White respondents. Next, in Model 3, the results show that after including all control variables, each additional ACE is associated with a 5% lower odds of past year dental care use (aOR =.95, CI = .93 –.97, p < .001). Notably, the results of the race coefficients flip directions after accounting for all control variables, with non-Hispanic Black respondents having higher odds of past-year dental care use (aOR = 1.21, CI = 1.07–1.36, p = 0.002), and the difference between Hispanic and non-Hispanic White respondents becoming positive, but not statistically significant. Upon further analysis, this results from a suppressor effect most strongly influenced by respondent income (see Appendix D). In this case, income acts as a suppressor variable because it is negatively correlated with being non-Hispanic Black or Hispanic and positively associated with dental care use. By controlling for income, the analysis removes the portion of variance that masked the positive association between race and dental care use, revealing the reversed direction of the coefficients.

**Table 2:** Multivariable Logistic Regression of Dental Care Utilization on ACEs.

	Model 1: Bivariate (N = 88,728)	Model 2: Race/Ethnicity Control (N = 88,728)	Model 3: With Controls (N = 88,728)
**Variables**	**aOR (95% CI)**	**aOR (95% CI)**	**aOR (95% CI)**
Number of ACEs	0.92 (0.90-0.93)***	0.92 (0.90-0.93)***	0.95 (0.93-0.97)***
*Race/Ethnicity*			
Non-Hispanic White (Reference)		—	—
Non-Hispanic Black		0.81 (0.74-0.90)***	1.21 (1.07-1.36)**
Hispanic		0.59 (0.52-0.66)***	1.15 (1.00-1.33)
*Age*			
18-24 (Reference)			—
25-34			0.69 (0.57-0.82)***
35-44			0.65 (0.53-0.79)***
45-54			0.68 (0.56-0.82)***
55-64			0.75 (0.61-0.91)**
65+			0.78 (0.65-0.95)*
*Sex*			
Female (Reference)			—
Male			0.65 (0.60-0.70)***
*Marital Status*			
Married (Reference)			—
Divorced or Separated			0.88 (0.78-0.98)*
Widowed			0.79 (0.70-0.90)***
Never Married			0.89 (0.78-1.01)
Member of an Unmarried Couple			0.80 (0.65-0.98)*
*Education*			
Less than High School (Reference)			—
High School Graduate			1.34 (1.15-1.56)***
Some College			1.55 (1.33-1.81)***
College Graduate			2.18 (1.86-2.55)***
*Child in Home*			
No (Reference)			—
Yes			0.98 (0.89-1.08)
*Veteran Status*			
No (Reference)			—
Yes			0.96 (0.86-1.07)
*Household Income*			
Less than $10,000 (Reference)			—
$10,000 - $14,999			1.10 (0.84-1.43)
$15,000 - $19,999			1.03 (0.82-1.29)
$20,000 - $24,999			1.41 (1.13-1.76)**
$25,000 - $34,999			1.55 (1.26-1.92)***
$35,000 - $49,999			1.78 (1.44-2.19)***
$50,000 - $74,999			2.39 (1.93-2.95)***
$75,000 or more			3.97 (3.20-4.92)***
*Health Insurance*			
No (Reference)			—
Yes			1.92 (1.70-2.18)***

*** p < 0.001, ** p < 0.01, * p < 0.05.

*Abbreviations*: ACEs = adverse childhood experiences; aOR = adjusted odds ratio; CI = confidence interval.

**Fig 1:**
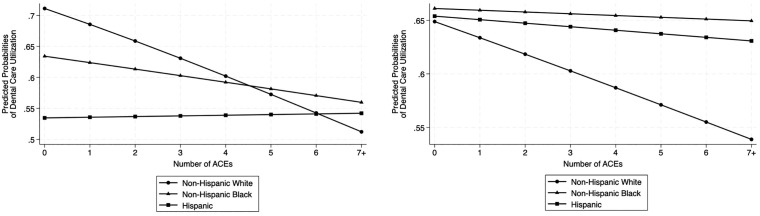
Predicted Probabilities of Past Year Dental Care Use on ACEs * Race/Ethnicity (without controls). B: Predicted Probabilities of Past Year Dental Care Use on ACEs * Race/Ethnicity (with controls).

Table 3 presents the multiplicative interaction term between the number of ACEs and race/ethnicity, demonstrating a positive and statistically significant interaction. To further explore this relationship, the predicted probabilities of the interaction term are visualized in Fig 1 [35]. For instance, the results in the bivariate model, provided in Fig 1, show that, at the level of 0 ACEs, there are substantial racial disparities in past-year dental care use: non-Hispanic White respondents have a predicted probability of.71 compared to.63 for non-Hispanic Black respondents and.53 for Hispanic respondents. However, a greater number of ACEs is associated with a lower predicted probability of dental care use for non-Hispanic White and non-Hispanic Black respondents, while the predicted probability remains relatively stable for Hispanic individuals. Notably, the decline in dental care use is steeper for non-Hispanic White respondents compared to non-Hispanic Black respondents. For instance, the predicted probability of past-year dental care use for non-Hispanic White respondents decreases from.71 at 0 ACEs to.51 at 7 + ACEs, representing a −28.0% change. In contrast, the decline for non-Hispanic Black respondents is from.63 to.56 over the same range, representing a −11.7% change. For Hispanic respondents, the results show an increase from.53 to.54, representing a + 1.4% change.

**Table 3:** Multivariable Logistic Regression of Dental Care Utilization on ACEs, with Interaction Term.

	Model 1: Bivariate (N = 88,728)	Model 2: With Controls (N = 88,728)
**Variables**		**aOR (95% CI)**
Number of ACEs	0.89 (0.87-0.90)***	0.93 (0.91-0.94)***
*Race/Ethnicity*		
Non-Hispanic White (Reference)	—	—
Non-Hispanic Black	0.70 (0.61-0.81)***	1.06 (0.91-1.25)
Hispanic	0.47 (0.40-0.55)***	1.03 (0.84-1.25)
Race/Ethnicity * Number of ACEs		
Non-Hispanic White * Number of ACEs (Reference)	—	—
Non-Hispanic Black * Number of ACEs	1.08 (1.03-1.14)**	1.07 (1.01-1.13)*
Hispanic * Number of ACEs	1.13 (1.07-1.20)***	1.06 (0.99-1.13)
*Age*		
18-24 (Reference)		—
25-34		0.69 (0.58-0.83)***
35-44		0.65 (0.54-0.79)***
45-54		0.68 (0.56-0.82)***
55-64		0.75 (0.61-0.91)**
65+		0.78 (0.64-0.94)**
*Sex*		
Female (Reference)		—
Male		0.64 (0.59-0.70)***
*Marital Status*		
Married (Reference)		—
Divorced or Separated		0.88 (0.78-0.98)*
Widowed		0.78 (0.69-0.89)***
Never Married		0.88 (0.78-1.00)
Member of an Unmarried Couple		0.81 (0.66-0.99)*
*Education*		
Less than High School (Reference)		—
High School Graduate		1.33 (1.14-1.54)***
Some College		1.54 (1.32-1.79)***
College Graduate		2.15 (1.84-2.52)***
*Child in Home*		
No (Reference)		—
Yes		0.97 (0.88-1.07)
*Veteran Status*		
No (Reference)		—
Yes		0.96 (0.87-1.07)
*Household Income*		
Less than $10,000 (Reference)		—
$10,000 - $14,999		1.10 (0.84-1.43)
$15,000 - $19,999		1.03 (0.82-1.29)
$20,000 - $24,999		1.41 (1.13-1.76)**
$25,000 - $34,999		1.54 (1.25-1.91)***
$35,000 - $49,999		1.77 (1.43-2.18)***
$50,000 - $74,999		2.37 (1.92-2.93)***
$75,000 or more		3.94 (3.17-4.88)***
*Health Insurance*		
No (Reference)		—
Yes		1.93 (1.71-2.19)***

*** p < 0.001, ** p < 0.01, * p < 0.05.

*Abbreviations*: ACEs = adverse childhood experiences; aOR = adjusted odds ratio; CI = confidence interval.

Fig 1b visualizes this relationship between the number of ACEs and dental care utilization, now accounting for controls. In this model, at 0 ACEs, there exist few racial disparities in past-year dental care use: non-Hispanic White respondents and Hispanic respondents both have a predicted probability of 0.65, compared to 0.66 for non-Hispanic Black respondents. For non-Hispanic Black and Hispanic respondents, we see that the predicted probability of past-year dental care use remains relatively stable as the number of ACEs increases. For example, the predicted probability decreases from 0.66 at 0 ACEs to 0.65 at 7 + ACEs, representing a −1.7% change, for non-Hispanic Black respondents. For Hispanic respondents, the predicted probability decreases from 0.65 at 0 ACEs to 0.63 at 7 + ACEs, representing a −3.5% change. In contrast, there is a noticeable decline in the predicted probability of past-year dental care use as the number of ACEs increases for non-Hispanic White respondents. Predicted probability drops from 0.65 at 0 ACEs to 0.54 at 7 + ACEs, representing a −16.9% change.

### Supplementary analyses

Appendix E explores the income suppressor effect on this multiplicative interaction term. In Model 1, when income is excluded as a control variable, there remains a positive and statistically significant interaction. The predicted probabilities of the interaction term are visualized in Appendix F. At 0 ACEs, the results show relatively small racial disparities in past-year dental care: non-Hispanic White respondents have a predicted probability of.67 compared to.65 for non-Hispanic Black respondents and.63 for Hispanic respondents. As the number of ACEs increases, the probability of past-year dental care utilization remains relatively unchanged for both non-Hispanic Black respondents and Hispanic respondents. Even so, a large decline in past-year dental care use is seen in non-Hispanic White respondents. Specifically, when going from 0 ACEs to 7 ACEs, the predicted probability of past-year dental care use for non-Hispanic White respondents drops from.67 to.53, representing a −20.8% change. For non-Hispanic Black respondents, there is a decline from.65 to.62, representing a −4.5% change. For Hispanic respondents, there is a slight decline from.63 to.62, representing a −1.1% change.

Appendix G reports separate multivariable logistic regression models examining the relationship between each ACE and past-year dental care use. The results illustrate statistically significant associations between household mental illness (aOR =.83, CI = .76 –.90, p < .001), household alcoholism (aOR =.83, CI = .77 –.90, p < .001), household illegal drug use (aOR =.85, CI = .76 –.95, p = .004), parent divorce or separation (aOR =.90, CI = .83 –.97, p = .009), household domestic violence (aOR =.88, CI = .81 –.97, p = .011), physical abuse (aOR =.87, CI = .80 –.95, p = .001), verbal abuse (aOR =.80, CI = .74 –.86, p < .001), and sexual abuse (aOR =.88, CI = .79 –.98, p = .016).

## Discussion

Using data on adults residing in 21 states and Washington, DC from the 2020 BRFSS, the results indicated that a higher number of ACEs was significantly associated with lower odds of dental care use in adulthood. These results align with findings from prior research [20–23]. Additionally, this study also identified racial heterogeneity in this relationship, with the negative association between ACEs and dental care use being most pronounced among non-Hispanic White respondents. In contrast, among non-Hispanic Black and Hispanic respondents, there was no effect of cumulative ACE exposure on dental care use. These findings align with recent research on dental care use during pregnancy among a sample of recent mothers from North Dakota and South Dakota, which found that the negative association between ACEs and dental care use was concentrated among White respondents [23]. However, that prior study was conducted among recent mothers in only two states with a largely White population.

The differential association of ACEs on dental care use across racial groups may be explained through the disadvantage saturation hypothesis, which posits that individuals already facing substantial structural and social adversities are less sensitive to the additive effects of additional adversities [15,24,25], such as ACEs. Non-Hispanic Black and Hispanic populations often experience structural barriers to dental care, including systemic racism, socioeconomic disadvantage, and reduced access to healthcare, which may limit their baseline utilization of dental care regardless of ACE exposure [26,27,36,37]. In contrast, non-Hispanic White individuals, who on average experience fewer structural barriers, may face a greater relative impact of ACEs on dental care utilization because these early life adversities represent a more pronounced deviation from their normative expectations of access and care [38,39].

The association between ACE exposure and reduced dental care utilization also highlights the need for research that unpacks the underlying mechanisms driving this relationship. Understanding whether lower engagement stems from psychological factors (e.g., dental anxiety, mistrust of providers), structural barriers (e.g., cost, transportation, competing life priorities), or the interplay of both is critical to designing public health programs that can improve dental care use and oral health for ACEs-exposed populations. Such insights could guide the development of clinical and community-based strategies that directly target identified barriers. For example, in dental clinics, this might involve integrating trauma-informed care protocols into routine practice [40,41], while in the community, outreach could occur through mobile dental units [42] and collaborations with trusted local organizations that work with ACEs-exposed populations to overcome access barriers to care.

### Limitations and future directions

There are limitations in the current study that can be expanded upon in future research. First, the BRFSS data are cross-sectional, which prohibits any causal inference of relationship between ACEs and dental care use. Moving forward, longitudinal research is needed to examine how ACEs influence dental care utilization over the life course, including the mediating roles of factors such as trust in healthcare providers, socioeconomic status, and oral health literacy. Second, the data relies on self-report responses, which may be subject to recall or social desirability bias, including reporting ACEs and dental care use. Third, while the BRFSS data are large, certain racial groups were not included in the analysis due to small cell sizes. Future studies should investigate the association between ACEs and dental care use among other populations, such as Native American or Asian American groups. Finally, the study was limited to examining the link between ACEs and dental care use. However, it would also be valuable for future research to examine how ACEs are associated with oral health conditions in adulthood, such as dental caries, periodontal diseases, and tooth loss. Strengths of this study include the use of a large, nationally representative dataset encompassing adults from multiple US states, the inclusion of a detailed measure of ACEs, and, to the best of the authors’ knowledge, the first analysis of racial heterogeneity in the relationship between ACEs and dental care utilization using representative data of US adults across multiple states.

## Conclusion

The results of this study, together with an accumulating body of evidence that suggests ACEs exposure harms dental care utilization over the life course [6–8]. Importantly, our findings reveal that the association between ACEs and dental care use is not uniform across racial and ethnic groups. The negative association was most pronounced among non-Hispanic White adults, while being attenuated or absent among non-Hispanic Black and Hispanic adults—a pattern consistent with the disadvantage saturation hypothesis, which posits that the marginal effect of additional adversities may be smaller among populations already experiencing substantial structural and social barriers to care. Moving forward, the findings of this study suggest the need for additional research, including the design and testing of community-based intervention programs to increase dental care use for those who may have high-ACE populations.

## Supporting information

S1 Appendix Sample selection flowchart. (DOCX)
