# Supplementary material for: Adverse childhood experiences and adult dental care utilization in the United States: Variation by race and ethnicity
Source: PLoS One. 2025 Sep 30;20(9):e0332880. doi: 10.1371/journal.pone.0332880 (PMC12483223; doi:10.1371/journal.pone.0332880)
Supplement: S1 Appendix — (DOCX) [file pone.0332880.s001.docx]

**Appendix A: Sample Selection Flowchart**

| Participated in 2020 BRFSS | *N* = 401,958  (54 States & Territories) |
| --- | --- |
|  | **↓** |
| Participated in the “Adverse Childhood Experiences” Module | *N* = 129,389  (22 States) |
|  | **↓** |
| Answered Questions on Adverse Childhood Experiences | *N* = 117,800  (22 States) |
|  | **↓** |
| Answered Questions on Oral Health | *N* = 116,694  (22 States) |
| Has Data on Control Variables | ↓ |
|  | *N* = 97,019  (22 States) |
| Respondent is Non-Hispanic White, Non-Hispanic Black, or Hispanic | ↓ |
|  | *N* = 88,728  (22 States) |

**Appendix B: Number of Respondents by State (N = 88,728)**

| **State** | **Number of Respondents** |
| --- | --- |
| Alabama | 3,375 |
| Arizona | 5,507 |
| District of Colombia | 2,013 |
| Florida | 6,009 |
| Georgia | 5,012 |
| Hawaii | 2,493 |
| Idaho | 3,522 |
| Iowa | 6,281 |
| Kentucky | 2,409 |
| Mississippi | 4,078 |
| Missouri | 5,853 |
| Montana | 4,064 |
| Nevada | 1,237 |
| North Dakota | 2,871 |
| Rhode Island | 3,192 |
| South Carolina | 2,349 |
| South Dakota | 3,774 |
| Texas | 5,713 |
| Utah | 7,172 |
| Virginia | 5,528 |
| Wisconsin | 3,228 |
| Wyoming | 3,048 |

**Appendix C: Definitions and Coding of Adverse Childhood Experience Questions**

| Prologue: I'd like to ask you some questions about events that happened during your childhood. This information will allow us to better understand problems that may occur early in life and may help others in the future. This is a sensitive topic and some people may feel uncomfortable with these questions. At the end of this section, I will give you a phone number for an organization that can provide information and referral for these issues. Please keep in mind that you can ask me to skip any question you do not want to answer. All questions refer to the time period before you were 18 years of age. | | | | |
| --- | --- | --- | --- | --- |
| **ACE Item** | **Question** | **Original Coding** | **Coding for Analysis** |  |
| Household Mental Illness | Did you live with anyone who was depressed, mentally ill, or suicidal? | 1 Yes  2 No  7 Don't Know/Not Sure  9 Refused | 0 No  1 Yes  *Don't Know/Not Sure or Refused recoded as missing |  |
| Household Alcoholism | Did you live with anyone who was a problem drinker or alcoholic? | 1 Yes  2 No  7 Don't Know/Not Sure  9 Refused | 0 No  1 Yes  *Don't Know/Not Sure or Refused recoded as missing |  |
| Household Illegal Drug Use | Did you live with anyone who used illegal street drugs or who abused prescription medications? | 1 Yes  2 No  7 Don't Know/Not Sure  9 Refused | 0 No  1 Yes  *Don't Know/Not Sure or Refused recoded as missing |  |
| Household Incarceration | Did you live with anyone who served time or was sentenced to serve time in a prison, jail, or other correctional facility? | 1 Yes  2 No  7 Don't Know/Not Sure  9 Refused | 0 No  1 Yes  *Don't Know/Not Sure or Refused recoded as missing |  |
| Parents Divorced or Separated | Were your parents separated or divorced? | 1 Yes  2 No  7 Don't Know/Not Sure  8 Parents not married  9 Refused | 0 No or not Married  1 Yes  *Don't Know/Not Sure or Refused recoded as missing |  |
| Household Domestic Violence | How often did your parents or adults in your home ever slap, hit, kick, punch or beat each other up? Was it… | 1 Never  2 Once  3 More than once  7 Don't Know/Not Sure  9 Refused | 0 Never  1 Once or more  *Don't Know/Not Sure or Refused recoded as missing |  |
| Physical Abuse | Not including spanking, (before age 18), how often did a parent or adult in your home ever hit, beat, kick, or physically hurt you in any way? Was it… | 1 Never  2 Once  3 More than once  7 Don't Know/Not Sure  9 Refused | 0 Never  1 Once or more  *Don't Know/Not Sure or Refused recoded as missing |  |
| Verbal Abuse | How often did a parent or adult in your home ever swear at you, insult you, or put you down? Was it… | 1 Never  2 Once  3 More than once  7 Don't Know/Not Sure  9 Refused | 0 Never  1 Once or more  *Don't Know/Not Sure or Refused recoded as missing |  |
| Sexual Abuse | (1) How often did anyone at least 5 years older than you or an adult, ever touch you sexually? Was it…  (2) How often did anyone at least 5 years older than you or an adult, try to make you touch them sexually? Was it…  (3) How often did anyone at least 5 years older than you or an adult, force you to have sex? Was it… | 1 Never  2 Once  3 More than once  7 Don't Know/Not Sure  9 Refused | 0 Never to all 3 questions  1 Once or more to any of the 3 questions  *Don't Know/Not Sure or Refused recoded as missing |  |

**Appendix D: Multivariable Logistic Regression of Dental Care Utilization on ACEs, Demonstrating Suppressor Effect**

|  | **Model 1: Without Income Control**  **(N = 88,728)** | **Model 2: With Income Control**  **(N = 88,728)** |
| --- | --- | --- |
| **Variables** | **aOR (95% CI)** | **aOR (95% CI)** |
| Number of ACEs | 0.94 (0.92-0.96)*** | 0.95 (0.93-0.97)*** |
| *Race/Ethnicity* |  |  |
| Non-Hispanic White (Reference) | — | — |
| Non-Hispanic Black | 1.03 (0.91-1.15) | 1.21 (1.07-1.36)** |
| Hispanic | 0.97 (0.85-1.10) | 1.15 (1.00-1.33) |
| *Age* |  |  |
| 18-24 (Reference) | — | — |
| 25-34 | 0.64 (0.54-0.76)*** | 0.69 (0.57-0.82)*** |
| 35-44 | 0.65 (0.54-0.79)*** | 0.65 (0.53-0.79)*** |
| 45-54 | 0.68 (0.57-0.82)*** | 0.68 (0.56-0.82)*** |
| 55-64 | 0.68 (0.56-0.83)*** | 0.75 (0.61-0.91)** |
| 65+ | 0.61 (0.51-0.74)*** | 0.78 (0.65-0.95)* |
| *Sex* |  |  |
| Female (Reference) | — | — |
| Male | 0.72 (0.67-0.78)*** | 0.65 (0.60-0.70)*** |
| *Marital Status* |  |  |
| Married (Reference) | — | — |
| Divorced or Separated | 0.64 (0.58-0.71)*** | 0.88 (0.78-0.98)* |
| Widowed | 0.60 (0.53-0.67)*** | 0.79 (0.70-0.90)*** |
| Never Married | 0.67 (0.59-0.75)*** | 0.89 (0.78-1.01) |
| Member of an Unmarried Couple | 0.65 (0.53-0.79)*** | 0.80 (0.65-0.98)* |
| *Education* |  |  |
| Less than High School (Reference) | — | — |
| High School Graduate | 1.58 (1.36-1.82)*** | 1.34 (1.15-1.56)*** |
| Some College | 2.07 (1.80-2.39)*** | 1.55 (1.33-1.81)*** |
| College Graduate | 3.55 (3.08-4.10)*** | 2.18 (1.86-2.55)*** |
| *Child in Home* |  |  |
| No (Reference) | — | — |
| Yes | 0.97 (0.89-1.07) | 0.98 (0.89-1.08) |
| *Veteran Status* |  |  |
| No (Reference) | — | — |
| Yes | 0.98 (0.88-1.10) | 0.96 (0.86-1.07) |
| *Household Income* |  |  |
| Less than $10,000 (Reference) | — | — |
| $10,000 - $14,999 | — | 1.10 (0.84-1.43) |
| $15,000 - $19,999 | — | 1.03 (0.82-1.29) |
| $20,000 - $24,999 | — | 1.41 (1.13-1.76)** |
| $25,000 - $34,999 | — | 1.55 (1.26-1.92)*** |
| $35,000 - $49,999 | — | 1.78 (1.44-2.19)*** |
| $50,000 - $74,999 | — | 2.39 (1.93-2.95)*** |
| $75,000 or more | — | 3.97 (3.20-4.92)*** |
| *Health Insurance* |  |  |
| No (Reference) | — | — |
| Yes | 2.30 (2.03-2.60)*** | 1.92 (1.70-2.18)*** |

*** p<0.001, ** p<0.01, * p<0.05

*Abbreviations*: ACEs = adverse childhood experiences; aOR = adjusted odds ratio; CI = confidence interval

**Appendix E: Multivariable Logistic Regression of Dental Care Utilization on ACEs, with Interaction Term and Suppressor Effect Illustrated**

|  | **Model 1: Without Income Control**  **(N = 88,728)** | **Model 2: With Income Control**  **(N = 88,728)** |
| --- | --- | --- |
| **Variables** |  | **aOR (95% CI)** |
| Number of ACEs | 0.92 (0.90-0.93)*** | 0.93 (0.91-0.94)*** |
| *Race/Ethnicity* |  |  |
| Non-Hispanic White (Reference) | — | — |
| Non-Hispanic Black | 0.89 (0.77 - 1.05) | 1.06 (0.91-1.25) |
| Hispanic | 0.82 (0.68 - 0.98)* | 1.03 (0.84-1.25) |
| *Race/Ethnicity * Number of ACEs* |  |  |
| Non-Hispanic White * Number of ACEs (Reference) | — | — |
| Non-Hispanic Black * Number of ACEs | 1.07 (1.02 - 1.13)** | 1.07 (1.01-1.13)* |
| Hispanic * Number of ACEs | 1.09 (1.03 - 1.16)** | 1.06 (0.99-1.13) |
| *Age* |  |  |
| 18-24 (Reference) | — | — |
| 25-34 | 0.65 (0.54 - 0.77)*** | 0.69 (0.58-0.83)*** |
| 35-44 | 0.65 (0.54 - 0.79)*** | 0.65 (0.54-0.79)*** |
| 45-54 | 0.68 (0.57 - 0.82)*** | 0.68 (0.56-0.82)*** |
| 55-64 | 0.68 (0.57 - 0.83)*** | 0.75 (0.61-0.91)** |
| 65+ | 0.61 (0.50 - 0.73)*** | 0.78 (0.64-0.94)** |
| *Sex* |  |  |
| Female (Reference) | — | — |
| Male | 0.72 (0.67 - 0.78)*** | 0.64 (0.59-0.70)*** |
| *Marital Status* |  |  |
| Married (Reference) | — | — |
| Divorced or Separated | 0.64 (0.58 - 0.71)*** | 0.88 (0.78-0.98)* |
| Widowed | 0.59 (0.53 - 0.67)*** | 0.78 (0.69-0.89)*** |
| Never Married | 0.67 (0.59 - 0.75)*** | 0.88 (0.78-1.00) |
| Member of an Unmarried Couple | 0.66 (0.54 - 0.80)*** | 0.81 (0.66-0.99)* |
| *Education* |  |  |
| Less than High School (Reference) | — | — |
| High School Graduate | 1.55 (1.35 - 1.79)*** | 1.33 (1.14-1.54)*** |
| Some College | 2.04 (1.77 - 2.36)*** | 1.54 (1.32-1.79)*** |
| College Graduate | 3.48 (3.02 - 4.01)*** | 2.15 (1.84-2.52)*** |
| *Child in Home* |  |  |
| No (Reference) | — | — |
| Yes | 0.97 (0.89 - 1.07) | 0.97 (0.88-1.07) |
| *Veteran Status* |  |  |
| No (Reference) | — | — |
| Yes | 0.99 (0.89 - 1.10) | 0.96 (0.87-1.07) |
| *Household Income* |  |  |
| Less than $10,000 (Reference) | — | — |
| $10,000 - $14,999 | — | 1.10 (0.84-1.43) |
| $15,000 - $19,999 | — | 1.03 (0.82-1.29) |
| $20,000 - $24,999 | — | 1.41 (1.13-1.76)** |
| $25,000 - $34,999 | — | 1.54 (1.25-1.91)*** |
| $35,000 - $49,999 | — | 1.77 (1.43-2.18)*** |
| $50,000 - $74,999 | — | 2.37 (1.92-2.93)*** |
| $75,000 or more | — | 3.94 (3.17-4.88)*** |
| *Health Insurance* |  |  |
| No (Reference) | — | — |
| Yes | 2.31 (2.04 - 2.60)*** | 1.93 (1.71-2.19)*** |

*** p<0.001, ** p<0.01, * p<0.05

*Abbreviations*: ACEs = adverse childhood experiences; aOR = adjusted odds ratio; CI = confidence interval

**Appendix F: Predicted Probabilities of Past Year Dental Care Use on ACEs * Race/Ethnicity (without income)**

**Appendix G: Multivariable Logistic Regression of Dental Care Utilization on Individual ACE Item (*N* = 88,728)**

| **Type of ACE** | **aOR** | **95% CI** |
| --- | --- | --- |
| Household Mental Illness | 0.83*** | (0.76 – 0.90) |
| Household Alcoholism | 0.83*** | (0.77 - 0.90) |
| Household Illegal Drug Use | 0.85** | (0.76 – 0.95) |
| Household Incarceration | 0.93 | (0.82 – 1.06) |
| Parents Divorced or Separated | 0.90** | (0.83 – 0.97) |
| Household Domestic Violence | 0.88* | (0.81 – 0.97) |
| Physical Abuse | 0.87*** | (0.80 – 0.95) |
| Verbal Abuse | 0.80*** | (0.74 – 0.86) |
| Sexual Abuse | 0.88* | (0.79 – 0.98) |

*** p<0.001, ** p<0.01, * p<0.05

*Abbreviations*: ACEs = adverse childhood experiences; aOR = adjusted odds ratio; CI = confidence interval
